# Outcomes of Ponte osteotomy combined with posterior lumbar interbody fusion for reconstruction of coronal and sagittal balance in degenerative scoliosis

**DOI:** 10.1186/s13018-023-04371-7

**Published:** 2023-11-28

**Authors:** Haoran Zhang, You Du, Yiwei Zhao, Yang Yang, Jianguo Zhang, Shengru Wang

**Affiliations:** Department of Orthopedic Surgery, Peking Union Medical College Hospital, Peking Union Medical College and Chinese Academy of Medical Sciences, 1St Shuai Fu Yuan, Dongcheng District, Beijing, 100730 China

**Keywords:** Degenerative scoliosis, Interbody fusion, Ponte osteotomy, Spinal deformity, Lumbar lordosis

## Abstract

**Purpose:**

The purpose of the study was to evaluate the clinical efficacy and safety of using Ponte osteotomy combined with posterior lumbar interbody fusion in the treatment of patients with degenerative scoliosis.

**Method:**

The medical records and imaging data of degenerative scoliosis patients in our department from 2013 to 2022 were retrospectively collected. A total of 47 patients were included, including 16 male patients and 31 female patients. The mean follow-up was 47.8 months. Whole spine X-rays in the standing position were performed on all patients preoperatively, postoperatively, and at the latest follow-up. The length of hospital stay, complications, operative duration, estimated blood loss, instrumented segment, fused segment, clinical scores, and various radiological indicators were recorded.

**Results:**

The coronal vertical axis improved from 3.1 ± 1.9 cm preoperatively to 1.2 ± 1.0 cm postoperatively with an average reduction of 1.9 ± 1.7 cm. The preoperative coronal Cobb angle was 18.1 ± 10.6°, the immediate postoperative Cobb angle was 6.6 ± 3.9°, and the Cobb angle at the last follow-up was 5.8 ± 3.7°. The sagittal vertical axis decreased from 5.6 ± 3.7 cm preoperatively to 2.7 ± 1.9 cm immediately after the operation and was well maintained at the last follow-up (3.1 ± 2.5 cm). Lumbar lordosis increased from 22.2 ± 10.2° preoperatively to 40.4 ± 8.3° postoperatively and 36.0 ± 8.8° at the last follow-up. The ODI score, VAS low back pain score, and VAS leg pain score were also improved to varying degrees.

**Conclusion:**

Ponte osteotomy combined with posterior lumbar interbody fusion can significantly improve coronal and sagittal plane deformities and postoperative functional scores in patients with adult degenerative scoliosis.

## Introduction

Scoliosis in adults may be the result of further progression of juvenile or adolescent idiopathic scoliosis, or it may be associated with the development of degenerative changes. The former is called adult idiopathic scoliosis, and the latter is called “de novo” scoliosis or degenerative scoliosis (DS) [1]. DS is not uncommon in elderly patients, with an estimated prevalence of approximately 6% in those over 50 years of age [2]. As the population ages, DS has plagued more and more people and brought a life and the financial burden on patients and their families. ADS is the most common form of adult spinal deformity, which is defined as the spinal deformity in a skeletally mature patient with a scoliotic angle of greater than 10 and without a history of adolescent idiopathic scoliosis during childhood and adolescence.

Surgery is indicated for severe low back pain or radicular symptoms that are refractory to conservative treatment. The current conventional surgical strategies include decompression alone, decompression combined with instrumented posterior spinal fusion, and posterior decompression and osteotomy combined with interbody fusion. For patients with mild deformity and slight instability, decompression alone may be an appropriate treatment option. However, in cases with severe deformity and spinal stenosis, decompression without secure fusion is directly associated with the risk of postoperative potential iatrogenic instability and deformity progression [3].

The restoration of lumbar physiological lordosis is more important than the correction of lumbar scoliosis because sagittal imbalance will lead to forward trunk tilt, flat back deformity, and thoracic kyphosis [4]. Unlike juvenile or adolescent idiopathic scoliosis, DS is often accompanied by advanced age, decreased bone mass, rigid and severe scoliosis, sagittal plane imbalance, and loss of lumbar lordosis (LL). Since Cloward first described posterior lumbar interbody fusion (PLIF), PLIF surgery has become the most popular and effective interbody fusion procedure [5]. PLIF consists of adequate decompression, complete discectomy, and spinal fusion. Although several novel interbody fusion techniques have been developed, the advantages of PLIF remain. PLIF can completely decompress the central spinal canal and bilateral nerve root canals, which is necessary to relieve low back pain and neurogenic claudication in elderly patients. In addition, PLIF can avoid damage to the vasculature, superior hypogastric plexus near the aortic bifurcation, psoas muscle, and lumbar plexus caused by oblique lumbar interbody fusion (OLIF), anterior lumbar interbody fusion (ALIF), or extreme/direct lateral interbody fusion (XLIF/DLIF) [6]. Unfortunately, the previous studies have shown that PLIF is less effective in reconstructing LL, especially compared with OLIF or ALIF [7–11]. Due to the anterior approach or anterolateral approach, OLIF and ALIF surgery can better release the intervertebral space and place larger and wider intervertebral cages. The large cage can generate a larger contact area with the upper and lower endplates, thereby reducing the pressure; furthermore, the cage can span the epiphyseal rings on both sides of the vertebral body to contact the cortical bone, which can provide more stability [11].

To further improve the effect of LL reconstruction, we added multilevel Ponte osteotomy on the basis of PLIF, allowing the entire facet joint to be resected. In our department, multilevel Ponte osteotomy is routinely performed in degenerative scoliosis cases to achieve the best correction in the coronal and sagittal planes. Ponte osteotomy is a surgical procedure developed by Alberto Ponte in 1987. To achieve an acceptable kyphotic correction, this procedure needs to include complete resection of the facet joints, ligamentum flavum, spinous process, and base of the lamina [12]. In the previous literature, it has been shown that multilevel Ponte osteotomy can obtain a correction effect similar to that of vertebral column resection and has the advantages of reducing operation time, blood loss, and perioperative complications [13].

The aim of the current study was to evaluate the clinical outcomes and radiographic correction of coronal and sagittal imbalance after Ponte osteotomy combined with PLIF in patients with DS.

## Methods

### Study design and patient population

This study is a retrospective cohort study that involved collecting medical record data and imaging data of DS from 2013 to 2022 in our department's clinical database. The minimum follow-up was 1 year. We were able to retrieve a total of 57 DS cases from the database. The inclusion criteria included the following: (1) patients with clinical manifestations of low back pain, leg pain, or intermittent claudication in whom conservative treatment was ineffective; (2) preoperative imaging data suggested that the apical vertebra of scoliosis was in the thoracolumbar or lumbar spine; (3) medical records, imaging, and follow-up data were complete; and (4) no previous history of idiopathic scoliosis. The exclusion criteria included the following: (1) Scoliosis found before the age of 18; (2) presence of spinal tumors, ankylosing spondylitis, spinal tuberculosis, history of spinal surgery, history of spinal trauma, and metabolic bone disease; (3) patients with missing medical records and follow-up data; and (4) follow-up less than 1 year. After screening according to the inclusion and exclusion criteria, a total of 47 patients were included in this study, including 16 male patients and 31 female patients. The mean age was 65.9 ± 8.3 years. The mean follow-up was 47.8 ± 23.8 months.

The study was approved by the ethics committee of our institution, and informed consent was obtained from the participating patients.

### Surgical procedure

The optimal entry point was determined according to the anatomical structure, and the titanium alloy pedicle screw was inserted. Taking L4-5 as an example, the surgeon would first use a rongeur to bite off the spinous process, then use an ultrasonic bone osteotome to resect half of the L4 lamina and bilateral inferior articular processes, and repeatedly use a pituitary rongeur and Kerrison rongeur to resect the bilateral L5 superior articular process, finally completing the L4-5 Ponte osteotomy. A size-15 surgical knife was used to make a small incision on the bilateral L4-5 intervertebral disk, and an ultrasonic bone osteotome, bone knife, reamer, and curette were repeatedly used to remove the L4-5 intervertebral disk and the upper and lower cartilage endplates. Two interbody fusion cages equipped with allograft bone were taken and placed into the intervertebral space from both sides to a suitable depth to complete L4-5 PLIF. After sufficient decompression, it is necessary to ensure that the dural sac has no obvious indentation, the bilateral nerve root has no tension, and the nerve root foramen is unobstructed. Two preshaped cobalt–chromium–molybdenum rods of appropriate length were prepared and connected with bilateral screws. Intraoperative fluoroscopy confirmed that the correction effect was satisfactory, and finally, all the nuts were tightened simultaneously and progressively on the two rods.

### Assessment of clinical and radiographic outcomes

The data included age, sex, body mass index (BMI), age-adjusted Charlson comorbidity index (aCCI) [14], osteopenia, length of hospital stay, complications, operative duration, estimated blood loss, blood transfusion, American Society of Anaesthesiologists (ASA) score, instrumented segment, fusion segment, upper instrumented vertebra, and lower instrumented vertebra.

The evaluation of clinical outcomes was based on the Oswestry Disability Index (ODI) [15], visual analog scale (VAS) low back pain score, and VAS leg pain score [16]. These questionnaires were considered effective measures for evaluating spinal deformities. The ODI was first designed in 1980 to assess low back pain disability and was subsequently revised in 2000. It has been widely used and validated for thoracic and lumbar spine pain. The evaluation of complications was based on the classification in the previous literature [17].

**Fig. 1:**
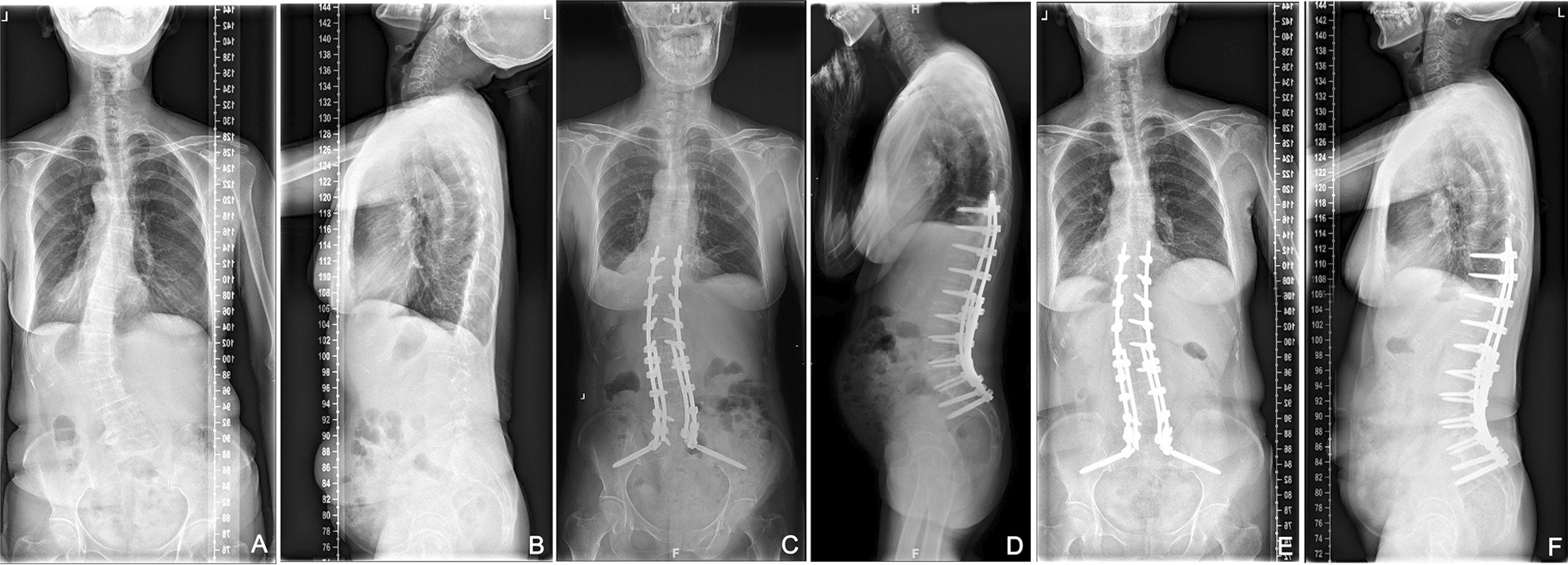
The imaging data of a typical case of degenerative scoliosis before operation and during the follow-up period. The patient is a 65-year-old female with clinical manifestations of low back pain and intermittent claudication. **A**, **B** The anteroposterior and lateral X-rays of the whole spine before operation. **C**, **D** The anteroposterior and lateral X-rays of the whole spine immediately after operation. **E**, **F** The anteroposterior and lateral X-rays of the whole spine after 5 years of follow-up. Comparing the imaging data, it could be found that Ponte osteotomy combined with PLIF technology can effectively reconstruct lumbar lordosis and restore coronal and sagittal plane balance in patients with degenerative scoliosis, and the effects of correction can be maintained for a long time after surgery

Radiographic outcomes were assessed preoperatively, postoperatively, and at the final follow-up (Fig. 1). Parameters included apical vertebral translation (AVT), coronal vertical axis (CVA), coronal Cobb angle, thoracolumbar kyphosis (TLK), LL, pelvic incidence (PI), pelvic tilt (PT), sacral slope (SS), pelvic incidence minus lumbar lordosis mismatch (PI‑LL), and sagittal vertical axis (SVA).

### Statistical analysis

Differences in preoperative and postoperative radiographic and clinical outcomes were measured using paired t-tests. *p* < 0.05 indicated a statistically significant difference. All statistics were completed using SPSS 27.0 software (IBM SPSS Inc., Chicago, Illinois, USA).

## Results

### Patient demographics and operative data

All patients had symptoms of low back pain, and some of them had neurogenic intermittent claudication (35 patients, 74.5%). The mean BMI was 24.9 ± 3.6 kg/m^2^. Thirty-five patients (74.5%) had one or more complications, and the mean aCCI index was 4.0 ± 1.5. According to the results of bone density measurement, 21 patients (44.7%) had osteopenia or osteoporosis. The total length of stay was 14.1 ± 5.4 days. The mean ASA grade of the 47 patients was 1.7 ± 0.9, the mean operative duration was 273.8 ± 69.4 min, and the mean estimated blood loss was 605.9 ± 464.1 ml. Among them, 27 patients (57.4%) required perioperative blood transfusion (Table 1).

**Table 1:** Characteristics of patients and procedures

Variable	Value
Age, years	65.9 ± 8.3
*Sex*	
Female	31 (34.0%)
Male	16 (66.0%)
BMI, kg/m^2^	24.9 ± 3.6
aCCI	4.0 ± 1.5
*Osteopenia/osteoporosis*	
Yes	21 (44.7%)
No	26 (55.3%)
Estimated blood loss, ml	605.9 ± 464.1
Operative duration, min	273.8 ± 69.4
ASA score	1.7 ± 0.9
Length of stay, days	14.1 ± 5.4
Follow-up, months	47.8 ± 23.8
*Blood transfusion*	
Yes	27 (57.4%)
No	20 (42.6%)
Fused segment	1.8 ± 0.8
Instrumented segment	5.6 ± 2.8
*Upper instrumented vertebra*	
T8-T10	8 (17.0%)
T11-L2	27 (57.4%)
L3–L4	12 (25.5%)
*Lower instrumented vertebra*	
L4	9 (19.1%)
L5	21 (44.7%)
Sacrum	17 (36.2%)

Values were expressed as number (%) or mean ± SD

BMI, body mass index and aCCI, age-adjusted Charlson comorbidity index

Across all procedures, the mean was 4.6 ± 1.8 for Ponte osteotomy and 1.8 ± 0.8 for fused segments. Among all surgical cases, 8 cases (17.0%) of upper instrumented vertebra were located at T8–T10, 27 cases (57.4%) at T11-L2, and 12 cases (25.5%) at L3–L4. The lower instrumented vertebra was located at L4 in 9 cases (19.1%), at L5 in 21 cases (44.7%), and at the sacrum in 17 cases (36.2%).

### Radiographic outcomes

Table 2 summarizes the results of preoperative and postoperative radiographic measurements in detail.

**Table 2:** Changes of imaging outcomes before and after surgery in patients with degenerative scoliosis

	Preoperative	Postoperative			Most recent follow-up^a^		
	Value (mean ± SD)	Value (mean ± SD)	Change	*p* value	Value (mean ± SD)	Change	*p* value
AVT (cm)	2.7 ± 2.2	1.2 ± 1.3	− 1.5 ± 1.3	< 0.001	1.2 ± 1.3	− 1.5 ± 1.4	< 0.001
CVA (cm)	3.1 ± 1.9	1.2 ± 1.0	− 1.9 ± 1.7	< 0.001	1.4 ± 1.0	− 1.6 ± 1.6	< 0.001
Coronal Cobb angle (°)	18.1 ± 10.6	6.6 ± 3.9	− 11.4 ± 8.8	< 0.001	5.8 ± 3.7	− 12.2 ± 8.9	< 0.001
TLK (°)	7.8 ± 7.2	7.1 ± 5.3	− 0.7 ± 3.9	0.252	7.2 ± 6.1	− 0.5 ± 5.2	0.490
LL (°)	22.2 ± 10.2	40.4 ± 8.3	18.2 ± 10.2	< 0.001	36.0 ± 8.8	13.8 ± 6.8	< 0.001
PT (°)	26.6 ± 9.2	20.6 ± 6.0	− 6.0 ± 6.4	< 0.001	18.0 ± 4.7	− 8.6 ± 7.7	< 0.001
SS (°)	28.5 ± 9.5	35.1 ± 7.3	6.5 ± 7.0	< 0.001	37.6 ± 7.6	9.1 ± 8.1	< 0.001
PI‑LL (°)	30.1 ± 12.8	15.3 ± 10.4	− 14.8 ± 9.4	< 0.001	19.6 ± 10.9	− 10.4 ± 6.1	< 0.001
SVA (cm)	5.6 ± 3.7	2.7 ± 1.9	− 3.0 ± 2.7	< 0.001	3.1 ± 2.5	− 2.6 ± 2.1	< 0.001

AVT, apical vertebral translation; CVA, coronal vertical axis; TLK, thoracolumbar kyphosis; LL, lumbar lordosis; PT, pelvic tilt; SS, sacral slope; PI‑LL, pelvic incidence minus lumbar lordosis mismatch; and SVA, sagittal vertical axis

^a^Compared to preoperative values

For the coronal plane, the mean preoperative AVT was 2.7 ± 2.2 cm, and the postoperative AVT was 1.2 ± 1.3 cm. The mean AVT was reduced by 1.5 ± 1.3 cm, and the difference was statistically significant (*p* < 0.001). The correction was not significantly lost at the recent follow-up (follow-up AVT = 1.2 ± 1.3 cm, p < 0.001). The mean preoperative CVA was 3.1 ± 1.9 cm, and the postoperative CVA was 1.2 ± 1.0 cm. The mean postoperative CVA decreased by 1.9 ± 1.7 cm, and the difference was statistically significant (*p* < 0.001). This improvement was maintained at the recent follow-up (follow-up CVA = 1.4 ± 1.0 cm, p < 0.001). The preoperative coronal Cobb angle was 18.1 ± 10.6°, the postoperative Cobb angle was 6.6 ± 3.9°, and the Cobb angle at the last follow-up was 5.8 ± 3.7°. The postoperative coronal Cobb angle (*p* < 0.001) and follow-up coronal Cobb angle (*p* < 0.001) were significantly improved compared with the preoperative condition.

For sagittal plane parameters, there was also a significant improvement in SVA. The SVA decreased from 5.6 ± 3.7 cm before the operation to 2.7 ± 1.9 cm after the operation (*p* < 0.001), and the correction was well maintained at the last follow-up (follow-up SVA = 3.1 ± 2.5 cm, *p* < 0.001). The mean TLK was 7.8 ± 7.2° preoperatively, 7.1 ± 5.3° postoperatively, and 7.2 ± 6.1° at the latest follow-up. There was no significant difference among the three groups (*p* = 0.252 and *p* = 0.490). The mean LL before the operation was 22.2 ± 10.2°, the postoperative LL was 40.4 ± 8.3°, and the LL at the last follow-up was 36.0 ± 8.8°. The LL was well reconstructed both immediately after the operation (mean difference was 18.2 ± 10.2°, *p* < 0.001) and at the last follow-up (mean difference was 13.8 ± 6.8°, *p* < 0.001).

Spinopelvic parameters were also significantly corrected. The mean PI was 55.7°, the PT decreased from 26.6 ± 9.2° preoperatively to 20.6 ± 6.0° postoperatively (mean difference was -6.0 ± 6.4°, *p* < 0.001), and correction was maintained during follow-up (follow-up PT = 18.0 ± 4.7°, mean difference was -8.6 ± 7.7°, *p* < 0.001). SS increased from 28.5 ± 9.5° before the operation to 35.1 ± 7.3° immediately after the operation, and no significant correction loss was found in follow-up (37.6 ± 7.6°). The PI‑LL also improved from 30.1 ± 12.8° preoperatively to 15.3 ± 10.4° postoperatively and 19.6 ± 10.9° at the last follow-up. These changes were statistically significant (*p* < 0.001).

### Clinical outcomes

ODI questionnaires were administered preoperatively, postoperatively, and at the final follow-up (Table 3). The mean ODI score postoperatively (29.2 ± 4.7) was considerably lower than the preoperative score (32.2 ± 6.1), and the difference was significant (mean difference was − 3.0 ± 5.0, *p* < 0.001); at the last follow-up, the mean ODI score (19.0 ± 6.0) was still significantly lower than the preoperative score, and the score decreased by 13.1 ± 7.1 (*p* < 0.001). Of the 47 patients, 31 (66.0%) patients had an improvement of more than 25% in the ODI score.

**Table 3:** Changes of ODI scores and VAS scores before and after surgery in patients with degenerative scoliosis

	Preoperative	Postoperative			Most recent follow-up^a^		
	Value (mean ± SD)	Value (mean ± SD)	Change	*p* value	Value (mean ± SD)	Change	*p* value
ODI	32.2 ± 6.1	29.2 ± 4.7	− 3.0 ± 5.0	< 0.001	19.0 ± 6.0	− 13.1 ± 7.1	< 0.001
Back pain (VAS)	4.9 ± 1.6	3.5 ± 1.3	− 1.4 ± 2.2	< 0.001	2.5 ± 1.4	− 2.4 ± 1.7	< 0.001
Leg pain (VAS)	2.7 ± 1.7	1.9 ± 1.2	− 0.9 ± 1.5	< 0.001	1.2 ± 1.3	− 1.6 ± 1.6	< 0.001

ODI, Oswestry Disability Index and VAS, visual analog scale

^a^Compared to preoperative values

The VAS low back pain score and VAS leg pain score also improved significantly postoperatively (Table 3). The mean VAS low back pain score was 4.9 ± 1.6 preoperatively and 3.5 ± 1.3 postoperatively (mean difference was − 1.4 ± 2.2, *p* < 0.001), and the VAS leg pain score at the recent follow-up was 2.5 ± 1.4 (mean difference was − 2.4 ± 1.7, *p* < 0.001). The mean VAS leg pain score was 2.7 ± 1.7 preoperatively and 1.9 ± 1.2 postoperatively (mean difference was − 0.9 ± 1.5, *p* < 0.001), and the VAS leg pain score at the recent follow-up was 1.2 ± 1.3 (mean difference was − 1.6 ± 1.6, *p* < 0.001). At the last follow-up, 61.7% (29 patients) and 23.4% (11 patients) of the patients had improved VAS low back pain scores and VAS leg pain scores by more than 25%, respectively.

### Complications

Of the 47 patients who underwent surgery, 17 patients (36.2%) experienced intraoperative or postoperative complications. No major medical complications (acute myocardial infarction, cerebrovascular accident, severe pneumonia, etc.) occurred during the perioperative period (Table 4).

**Table 4:** Complications in patients undergoing posterior lumbar interbody fusion and Ponte osteotomy

Complication	Number of patients (%)
Superficial wound infection	2 (4.3%)
Cerebrospinal fluid leakage	2 (4.3%)
Neurological deficits	3 (6.4%)
Liver damage	1 (2.1%)
Aspiration pneumonia	1 (2.1%)
Urinary tract infection	1 (2.1%)
Deep vein thrombosis of lower limb	1 (2.1%)
Nonunion requiring revision	1 (2.1%)
Instrumentation complication requiring revision	3 (6.4%)
Adjacent segment degeneration requiring revision	2 (4.3%)
Total	17/47 (36.2%)

Superficial wound infection occurred in two patients, one of whom was cured by wound dressing change combined with antibiotic treatment; the other underwent bedside debridement and finally healed well. Two patients had intraoperative or perioperative cerebrospinal fluid leakage. For one patient, the cerebrospinal fluid leak was plugged with absorbable adhesive membrane and gelatin sponge during the operation. All patients were treated with intermittent tube clamping after the operation, and all recovered well. Three patients developed symptoms of neurological deficits, manifested as transient exacerbation of low back and leg pain, transient decrease in muscle strength, or hypoesthesia. All patients improved after receiving high-dose corticosteroids, neurotrophic and dehydration therapy, and the last follow-up showed that neurological deficits had almost disappeared. Urinary tract infection occurred in one patient after the operation, and urinary tract irritation disappeared after prolonged intravenous infusion of antibiotics. One patient had unexplained elevated liver enzymes 3 days after the operation, which was considered related to drug-induced liver injury, and the liver enzymes returned to normal after liver-protecting drugs were added. One patient experienced hypoxemia and shortness of breath after the operation, and imaging examinations suggested atelectasis and pneumonia. The condition gradually improved after regular use of exercise training in pulmonary rehabilitation. One patient was found to have a progressive increase in the value of D-dimer, and deep vein ultrasound of the lower extremities indicated intermuscular venous thrombosis. After the drainage tube was extracted, we gave the patient oral anticoagulant treatment. In addition, six patients underwent revision surgery due to issues such as screw loosening, nonunion, or adjacent segment degeneration, and postoperative follow-up showed good recovery.

## Discussion

DS is a disease that severely reduces the quality of life and can even be disabling. The primary goals of DS surgery are adequate decompression, stabilization of the spine, and restoration of coronal and sagittal balance.

The previous studies have shown an association between coronal and sagittal imbalances and the severity of clinical symptoms in patients with DS [18, 19]. The overall balance of the spine reduces the energy required to walk, reduces pain and fatigue during walking, and improves appearance and patient satisfaction [20]. Glassman followed up on the imaging data and functional scores of 298 patients with adult scoliosis and found that the coronal and sagittal plane balance is the most reliable predictor of discomfort symptoms, especially the sagittal parameters [18]. They suggest that restoration of normal sagittal balance is a key goal of corrective surgery for DS, and that the degree of correction of coronal deformity appears to be a less important parameter. We adopted the method of posterior Ponte osteotomy, which not only avoided the risks of intestinal injury, vascular injury, or retrograde ejaculation that may accompany anterior surgery but also obtained good immediate coronal and sagittal plane correction outcomes.

One of the main manifestations of DS is the loss of LL. The reduction in LL will lead to a mismatch of PI‑LL, which will cause an increase in pressure in the intervertebral space and facet joints and finally cause trunk shift and muscle fatigue. The previous studies have shown a significant positive correlation between LL and postoperative quality of life [19]. Schwab prospectively reviewed the spino-pelvic parameters and functional scores of 492 adult patients with spinal deformities, and the results suggested that PI‑LL > 11° was an accurate predictor of severe functional impairment (ODI > 40) [21].

The traditional PLIF procedure is to perform a laminotomy medial to the facet joint and retract the dura to expose a corridor to the disk space. However, PLIF may pose difficulties in correcting coronal imbalances and restoring lordosis. Endplate preparation in PLIF is difficult compared to anterior fusion approaches [11]. To compensate for the poor effect of PLIF on LL reconstruction, we additionally performed multilevel Ponte osteotomies. Multilevel Ponte osteotomies are mainly used for resection of facet joints, lamina, and ligamentum flavum and generally involve 1–2 segments less than the instrumented segments. The data showed that the LL can be increased by 18.2° and 13.8° at the immediate postoperative and final follow-up, respectively. On average, this hybrid technique restores approximately 10° of LL perfused segment. This effect compares favorably with the anterior 4.5° and lateral 2.2° values reported in the literature, let alone the transforaminal 0.8° [8].

It must be acknowledged that this study has some limitations. First, this study is a retrospective cohort study, and it is difficult to completely avoid recall bias and selection bias. Second, we did not compare the pros and cons of PLIF and other lumbar interbody fusion techniques simultaneously in one cohort. In the future studies, we will compare the clinical and radiological differences between PLIF and OLIF/ALIF.

## Conclusions

The results showed that patients received good coronal and sagittal correction effects, and this effect was well maintained in the subsequent follow-up. We believe that the application of Ponte osteotomy combined with PLIF for spinal correction in DS in patients is effective and safe.

## Data Availability

The datasets used and/or analyzed during the current study are available from the corresponding author on reasonable request.
